# Conformation-dependent recognition of HIV Gp120 by DARPins provides novel possibilities to develop distinct HIV entry inhibitors

**DOI:** 10.1186/1742-4690-9-S2-P218

**Published:** 2012-09-13

**Authors:** AM Mann, N Friedrich, A Krarup, P Rusert, J Weber, B Dreier, P Pugach, M Robbiani, JA Robinson, A Pluckthun, A Trkola

**Affiliations:** 1 University of Zurich, Zurich, Switzerland; 2 Institute of Biochemistry, University of Zurich, Zurich, Switzerland; 3 Center for Biomedical Research, Population Council, New York, NY, USA; 4 Institute of Organic Chemistry, University of Zurich, Zurich, Switzerland

## Background

Designed Ankyrin Repeat proteins (DARPins) are a novel type of binding protein scaffold designed as antibody alternative for biomedical applications. DARPins share many properties with antibodies, most noteworthy a high target specificity and affinity, but they differ from antibodies in size, structure and binding pattern and, importantly, favorable biophysical properties such as exceptional stability. This together with high-yield prokaryotic production renders DARPins promising candidates for microbicide development.

## Methods

DARPin DNA libraries encoding either two or three internal ankyrin repeats were subjected to ribosome display selections and panned against recombinant gp120 proteins. Wild type, CD4 induced, V1V2 truncated and deglycosylated JR-FL gp120, as well as structural V3 loop mimetics were probed as target. Obtained DARPin clones were sequenced, mapped for reactivity with HIV gp120 by ELISA and probed for inhibitory activity using the TZM-bl pseudotype virus inhibition assay.

## Results

DARPin selection proved more successful when conformationally arrested targets were used for panning. Overall, gp120-specific DARPins recognizing a variety of epitopes including the CD4bs, CD4i and the V3 loop were obtained. Gp120 mutant binding analysis revealed that DARPin molecules depended to a higher degree on a structural conservation of the envelope protein than gp120 specific antibodies recognizing overlapping domains. Most noteworthy, V3 loop specific clones were selected, which unlike V3 loop antibodies, recognized the V3 loop in a conformation-dependent manner and thus do not efficiently bind linear V3 loop peptides. In contrast to V3-loop directed antibodies these DARPins proved to bypass the envelope shielding by the V1V2 domain and thus were capable of neutralizing TIER-2 viruses.

## Conclusion

Gp120 proved a challenging target for selection of DARPin binders against gp120. Nonetheless, by combining different selection strategies we were able to derive a variety of gp120-specific DARPin molecules, including some with unique HIV entry blocking activity.

